# A Theoretical and Experimental Study to Optimize Cell Differentiation in a Novel Intestinal Chip

**DOI:** 10.3389/fbioe.2020.00763

**Published:** 2020-07-24

**Authors:** Nicky Langerak, Haysam M. M. Ahmed, Yang Li, Igor R. Middel, Hossein Eslami Amirabadi, Jos Malda, Rosalinde Masereeuw, René van Roij

**Affiliations:** ^1^Institute for Theoretical Physics, Utrecht University, Utrecht, Netherlands; ^2^Division of Pharmacology, Institute for Pharmaceutical Sciences, Utrecht University, Utrecht, Netherlands; ^3^Utrecht Biofabrication Facility, University Medical Center Utrecht, Utrecht, Netherlands; ^4^Human Cell Biology Group, Division of Metabolic Health Research, TNO, Zeist, Netherlands

**Keywords:** gut-on-chip, shear stress, cell differentiation, 3D printing, numerical computation

## Abstract

Microphysiological systems have potential as test systems in studying the intestinal barrier, in which shear stress is critical for the differentiation of Caco-2 cells into enterocytes. The most commonly used *in vitro* gut model for intestinal barrier studies is based on trans-well cultures. Albeit useful, these culture systems lack physiological shear stress which is believed to be critical for the differentiation of Caco-2 cells into enterocytes and to form tight monolayers. Conversely, organ-on-chip models have presented themselves as a promising alternative since it provides cells with the required shear stress. To this end, a novel biocompatible 3D-printed microfluidic device was developed. In this device, Caco-2 cells were seeded under physiologically-relevant unidirectional shear stress and compared to cells cultured under gravity-driven flow. Using numerical studies, the flow rate that corresponds to the required shear stress was calculated. Experimental tests were conducted to verify the effect of this on cell differentiation. The experiments clearly showed an enhancement of cell differentiation potential in a unidirectional physiologically-relevant pump-driven flow system (PDFS) as opposed to the simpler bidirectional gravity-driven flow system (GDFS). Additionally, computational modeling of an adapted design confirmed its ability to supply all cells with a more homogeneous shear stress, potentially further enhancing their differentiation. The shear stress in the adapted design can be well-approximated with analytic methods, thus allowing for efficient predictions for all parameter values in the system. The developed novel microfluidic device led to the formation of a tighter monolayer and enhanced functional properties of the differentiated Caco-2 cells, which presents a promising tool for preclinical *in vitro* testing of drugs in an animal-free platform.

## 1. Introduction

The small intestine plays a key role in the selective absorption of nutrients and drugs, and forms the first line of defense via its epithelial barrier. Three-dimensional (3D) experimental set-ups that can mimic human intestinal micro-environments may be instrumental in studying absorption processes and intestinal barrier integrity, which is now widely addressed by the so-called organ-on-a-chip technology. These chips aim to mimic the human organ physiology by replicating functional parts of organs in a controlled laboratory environment.

Microphysiological systems with biomimicry micro-architectures are usually fabricated from an optically clear plastic, such as polydimethylsiloxane (PDMS) and contain perfused hollow microchannels inhabited by living human cells as described by Bhatia and Ingber (2014). As a result of this structure, they allow for high-resolution, real-time imaging of biochemical activities in living human cells in a functional, yet controlled, organ context as described by Sontheimer-Phelps et al. (2019).

Various microphysiological systems have been developed that mimic mechanical, structural, and functional properties of the human small intestine (see e.g., Kim and Ingber, 2013; Trietsch et al., 2017; Kasendra et al., 2018; Jochems et al., 2019). These devices have already displayed the capability to reproduce organ-level responses and may be transferred to pharmaceutical industries to be implemented in preclinical research and have potential to replace animal studies for e.g., drug screening.

As carefully reviewed by Sontheimer-Phelps et al. (2019), however, microphysiological systems still have their limitations. One of these is the materials used to fabricate the device; PDMS is for instance known to non-specifically absorb small molecules, including certain drugs (Li et al., 2009; Su et al., 2011). In this work, we present a newly designed microfluidic device to study drug transport and metabolic functions of initially the intestine, with the potential to study other organs at a later stage. The experimental setup is constructed using an inert biocompatible polylactic acid (PLA) instead of the common microfabrication with PDMS to minimize solute absorption. In a comparison study, PDMS majorly absorbed hydrophobic small molecules, while PLA absorbed neither hydrophobic nor hydrophilic small molecules (Ongaro et al., 2020). Furthermore, we used a hollow fiber membrane set-up as in Jochems et al. (2019) to make the system compatible with transepithelial transport studies. The design of the microfluidic chip was meant to optimize cell differentiation toward a tight monolayer of functional organ cells. To enhance cell differentiation, we aim for a tuneable, time oscillating and homogeneous shear stress on the cells in the physiologically relevant regime of 0.002−0.8dyne/cm^2 for intestinal cells (Olesen et al., 1988; Lentle and Janssen, 2008; Ishikawa et al., 2011). As a secondary optimization condition, we aim to achieve this shear stress at a low flow rate to allow for solute diffusion and drug transport in our experiments.

**Figure 1:**
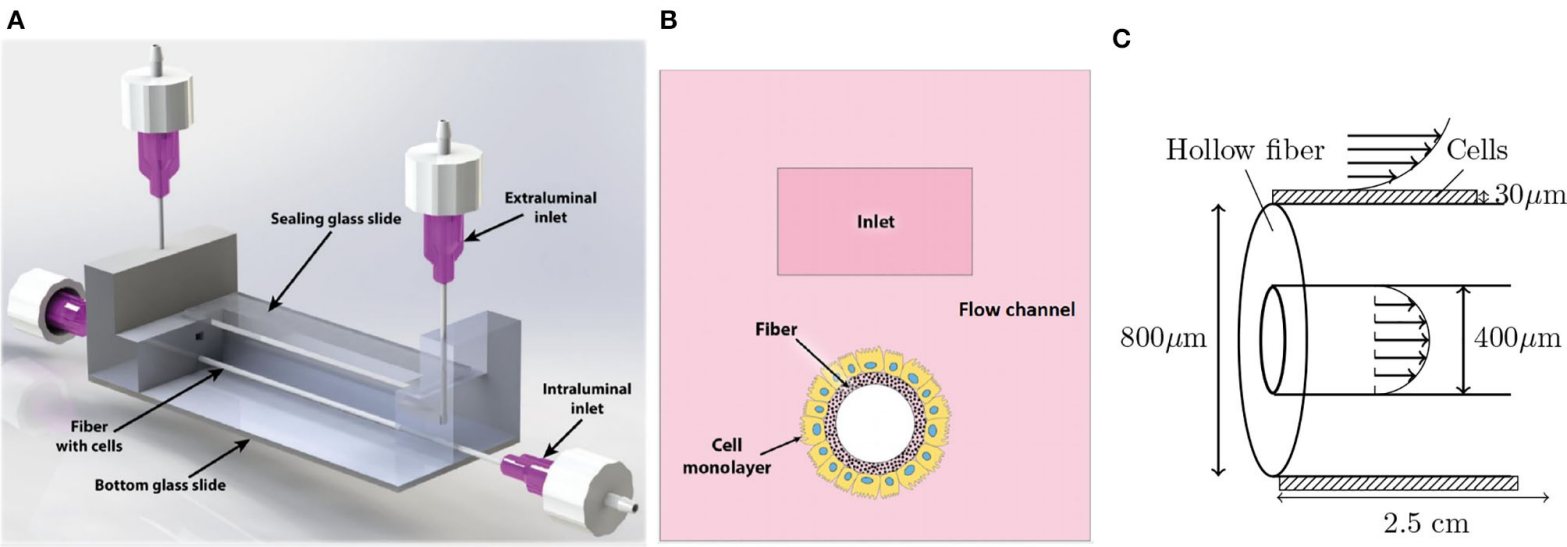
Overview of the experimental setup. **(A)** The microfluidic chip with the in- and outlets as well as the fiber and transparent glass top and bottom. Fluid flow is driven by a peristaltic pump that is separately connected to the intra- and extraluminal inlets and outlets. **(B)** Cross section of the microfluidic chip. **(C)** Detailed view of the fiber and cells. The patterned domain indicates the cell layer, on which the fluid exerts a shear stress. The fluid velocity is indicated with the arrows inside and around the fiber. The steady flow rates in this device will be of order *Q*_0_ = 125μL/min. Note that the figures are not to scale.

In Figure 1A, we show an overview of the experimental setup. The fluid flow in the setup is driven by a peristaltic pump, with an approximate harmonic pressure gradient as a result. This aims to mimic the time-dependent nature of fluid flow in human organs. The pump uses two reservoirs to independently perfuse two types of medium through side and top pathways, representing the intra- and extraluminal fluids, respectively. An off-scale cross-section of the system is shown in Figure 1B, which indicates that a perfusable hollow fiber is inserted into the flow channel. Cells were seeded on the outside of this fiber and shear stress, induced by the flow, will lead to the cell differentiation into a tight and, functional monolayer. A more detailed schematic with the system size and flow around the cells and fiber is shown in Figure 1C. Note that a fluid flow through the fiber was implemented as well to ensure a small pressure difference between the in- and outside of the fiber walls.

To find an optimal design in terms of cell differentiation, our methods can be divided in three steps. To give the reader a clear overview, we briefly discuss these steps before we go into detail on the methodology. First, numeric calculations on the flow velocity and shear stress were performed in potential devices, to find a simple design that gave shear stresses in the physiological relevant regime. Second, experiments were performed in the resulting experimental design (Figure 1) to quantitatively verify the calculations and measure the cell differentiation. Finally, as the third step, we investigated further optimization options in the design using both numerical and analytic calculations.

## 2. Materials and Methods

### 2.1. Chamber Fabrication

The device is constructed through Fused Deposition Modeling (FDM) 3D printing with Ultimaker 3 (Ultimaker B.V.), which extrudes and selectively deposits thermoplastic (Polylactic acid, PLA) filament in the pre-determined position in a layer-by-layer manner. This fabrication process allows for efficient reflection of theoretical studies into prototypes. The 3-dimensional printable file was first designed in SolidWorks 2018 (Solidworks Corp.) and exported to G-code file via Ultimaker Cura (Ultimaker B.V.). The layerwise G-code file was thus read by the 3D printer and printed through a 0.4 mm nozzle in a layer-by-layer manner at a layer resolution of 60 microns. For the convenience of imaging, the top and bottom surfaces of the printed devices were partially open and sealed with cover slips, respectively.

In a typical chamber with the dimension of 75 mm (length) by 12.5 mm (width) by 3.65 mm (height), a open inner liquid reservoir of 25.1 mm (length) by 3 mm (width) sits in the very center of the chamber. Two flow pathways (as in Figure 1B), i.e., tubular channel (1.47 mm diameter) and cuboid channel (1.3 by 0.7 mm), are separated by 0.4 mm along the thickness direction of the chamber.

### 2.2. Chamber Assembly

SENUOfil type H-MF-0.2 hollow fiber capillary membranes (SENUOFIL, Tianjin, China) were fitted into the 3D-printed chambers and fixed in place with a high-viscosity dental glue “GI-MASK” (Coltene, Altstätten, Switzerland). Glass cover slips were used to seal the top and bottom of the chamber and fixed in place using medical-grade epoxy glue “Loctite Hysol M-31CL” (Henkel adhesives, Bopfingen, Germany). 18g syringe needles were used for intraluminal compartment.

### 2.3. Hollow Fiber Membrane Coating and Seeding

All chemicals were purchased from Sigma-Aldrich (Zwijndrecht, The Netherlands) unless stated otherwise. Following the assembly, the chambers were decontaminated in 70% (v/v) EtOH for 30 min. Next, chambers were washed with phosphate-buffered saline (PBS) twice and the hollow fiber membranes (HFM) were coated with filter-sterilized L-3,4-di-hydroxy-phenylalanine (L-Dopa, 2 mg/mL in 10 mM Tris buffer, pH 8.5). L-Dopa has been widely used in tissue engineering, and is known to be enriched in mussel-like adhesive proteins (Lee et al., 2009) and can adhere to a wide range of surfaces. L-Dopa self-polymerizes and exploits the reactivity of the film to covalently bind the biomolecule Coll I on the membrane surface to form an extracellular matrix (Oo et al., 2011). The fibers were then incubated at 37°C for 4 h to allow full polymerization of L-Dopa. After drying at 37°C for 5 min, HFM were washed with PBS and put in human collagen I solution (25 μg/mL in PBS) for another incubation at 37°C for 2 h. HFM were dried for 5 min at 37°C and unbound human collagen I was washed off with PBS. Finally, HFM were washed and stored in PBS awaiting cell seeding for a maximum of 4 days. Caco-2 cells (ATCC, Wesel, Germany) passages between 70 and 80 were washed, trypsinized and resuspended in culture medium [high glucose Fluorobrite medium (Gibco) with fetal calf serum (10% v/v), Glutamax (1%v/v), MEM Non-Essential Amino Acids (1%v/v), and penicillin/streptomycin (1% v/v)] at a concentration of 10^6^ cells/ml; hereafter referred to as cell suspension. For cell seeding, 0.25 mL cell suspension was added per channel and incubated at 37°C, 5% CO_2_ for 4 h in static conditions to allow cell adherence.

### 2.4. Flow Experiments

Following initial seeding, chambers were either exposed to passive gravity-driven flow for a period of 21 days or active pump-driven flow for 10 days. In the gravity-driven flow system (GDFS), flow was achieved by means of a 2-dimensional rocking platform (VWR, Breda, The Netherlands) with a speed rate of six rotations per minute at an angle of 10°, as described previously by Jochems et al. (2019). As for the pump-driven flow system (PDFS), flow rates of 30, 60, 90, and 125 μL/min were tested. Medium was refreshed every 2–3 days.

### 2.5. Cell Counting

Cell counting for normalization of activity per cell number was carried out utilizing the Cell Counting Kit (CCK-8) according to the manufacturer's protocol. Briefly, cell suspensions were seeded in 12-well plates and incubated at 37°C, 5% CO_2_ for 24 h. Subsequently, the medium was removed and replaced with the CCK-8 reagent diluted 1:10 in culture medium. The plate was incubated for 4 h at 37°C, 5% CO_2_, after which absorbance was measured at 450 nm with iMARK™microplate absorbance reader (Biorad, Veenendaal, Netherlands). Cell number was calculated using a standard curve prepared using a serial dilution of Caco-2 cells.

### 2.6. p-Cresol Metabolism

To assess the metabolic capacity of the differentiated Caco-2 cells, p-cresol was used as a model compound. After culturing the Caco-2 cells for 3 weeks in the GDFS, p-cresol was added either at a concentration of 50 μM for 3 and 6 h, or 100 μM for 12 and 24 h. The effect of p-cresol and its metabolites on the monolayer tightness was assessed using FITC-inulin leakage as described later. Based on the results of the initial experiment, exposure to p-cresol at a concentration of 50 μM for 3 h was used for all subsequent experiments.

To investigate the effect of 1,25-dihydroxyvitamin D3 (Vit D3) on the cell membrane integrity and the metabolic function of Caco-2 cells upon p-cresol treatment, cells in the GDFS were pre-treated with 100 nM Vit D3 for 24 h after 21 days in culture. 50 μM p-Cresol was then added for 3 h. Samples were then collected and analyzed using an ultra-high performance liquid chromatography mass-spectrometry (UHPLC-MS) method, as described by Mutsaers et al. (2011) to determine the concentration of the p-cresol metabolites p-cresol sulfate and p-cresol glucuronide.

To assess the effect of physiologically-relevant flow in the PDFS on p-cresol metabolism, both GDFS and PDFS were treated with 100 nM Vit D3 for 24 h after 21 and 10 days in culture, respectively. After which they were exposed to 50 μM p-cresol for 3 h. Samples were then collected and analyzed.

### 2.7. Immunofluorescent Staining

To investigate epithelial-layer integrity, phenotypical and morphological characteristics, immunostainings were performed. Cells were fixed using 2% paraformaldehyde for 15 min and then permeabilized using 0.3% (v/v) Triton X-100 in HBSS for 5 min. To prevent non-specific binding of antibodies, blocking was performed using 2% (w/v) bovine serum albumin (BSA) fraction V and 0.1% (v/v) Tween-20 in HBSS for 1 h. Fixed cells were incubated with primary antibodies, diluted in block solution, used were tight junction protein, zonula occludens-1 (ZO-1, 1:400) (Thermo Fisher Scientific, Bleiswijk, The Netherlands), enterocyte marker, Villin (1:400) (SCBT, Texas, USA) for 4 h. This was followed by incubation with goat-anti-mouse Alexafluor 488 (1:1,000; Thermo Fisher Scientific) or goat-anti-rabbit CF640R (1:1,000) as secondary antibodies. Finally, HFM were mounted using Prolong gold containing DAPI (Cell signaling technology, Leiden, The Netherlands) for nuclei staining. Images were acquired using the Leica TCS SP8 X (Leica Biosystems, Amsterdam, The Netherlands).

### 2.8. Leakage Assay

To assess monolayer integrity, an inulin-FITC (0.1 mg/ml in PBS) leakage assay was performed, as described in Jochems et al. (2019). The intraluminal inlet of the chamber was connected to warm inulin-FITC reservoir via a peristaltic pump (Ismatec Reglo, Cole parmer, Wertheim, Germany). The HFM was then perfused with inulin-FITC with a molecular weight of 3–6 kDa at a flow rate of 0.1 mL/min for 10 min. Samples were taken from the apical compartment. Fluorescence of the samples was measured at excitation wavelength of 492 nm and emission wavelength of 518 nm using Tecan infinite M200PRO plate reader (Tecan Austria GmbH).

The values of the apparent permeability *P*_*app*_ (in cm/s), which indicate the leakage of inulin-FITC, were calculated according to:

(1) $$\begin{array}{r} P_{app}=\frac{\frac{\text{d}Q}{\text{d}t}}{AC_{0}} \end{array}$$

where d*Q*/d*t* indicates the appearance rate of inulin-FITC over time (relative fluorescence unit/s), *A* is the surface area of the exposure area and *C*_0_ is the initial concentration of the warm inulin-FITC reservoir. As a control, coated-fibers without cells were used to determine leakage through the fiber which was then set to 100% leakage for further calculations.

### 2.9. Alkaline Phosphatase Activity

Alkaline phosphatase (ALP) activity was measured as enterocyte differentiation marker, using Amplite™Colorimetric Alkaline Phosphatase assay kit (AAT Bioquest, Sunnyvale, USA). The assay was performed according to the manufacturer protocol. Briefly, HFM were incubated with the pNPP substrate solution (diluted 1:1 in culture medium) for 15 min at 37°C; thereafter, absorbance was measured at 400 nm with iMARK™microplate absorbance reader (Biorad, Veenendaal, the Netherlands).

### 2.10. Statistical Significance

Every experiment was at least performed in triplicate, results are depicted as mean ± standard error of the mean. Statistical analysis was performed in Graphpad version 7 using either one-way ANOVA followed by a Dunnett's test, Student's *T*-test or Unpaired *T*-test (where applicable). A *P*-value of *p* < 0.05 was considered significantly different.

### 2.11. Governing Equations

We determined the flow around the cells theoretically with the standard Stokes equations, by solving

(2) $$\begin{array}{r} \rho \frac{\partial \text{u}}{\partial t}=-\nabla p+\eta \nabla^{2}\text{u},\;\nabla \cdot \text{u}=0 \end{array}$$

for the velocity profile **u**(**r**, *t*) and the pressure *p*(**r**, *t*) at position **r** and time *t*. In Equation (2), ρ = 997.8kg/m^3 and η = 0.78mPa s are the mass density and dynamic (shear) viscosity of the fluid, respectively. Note that ρ(**r**, *t*) = ρ is a spatial and temporal constant, since we assume an isotropic and incompressible medium. Furthermore, we have no external forces on our fluid and we restricted attention to the low-Reynolds number regime, where the flow is laminar without any turbulence.

**Figure 2:**
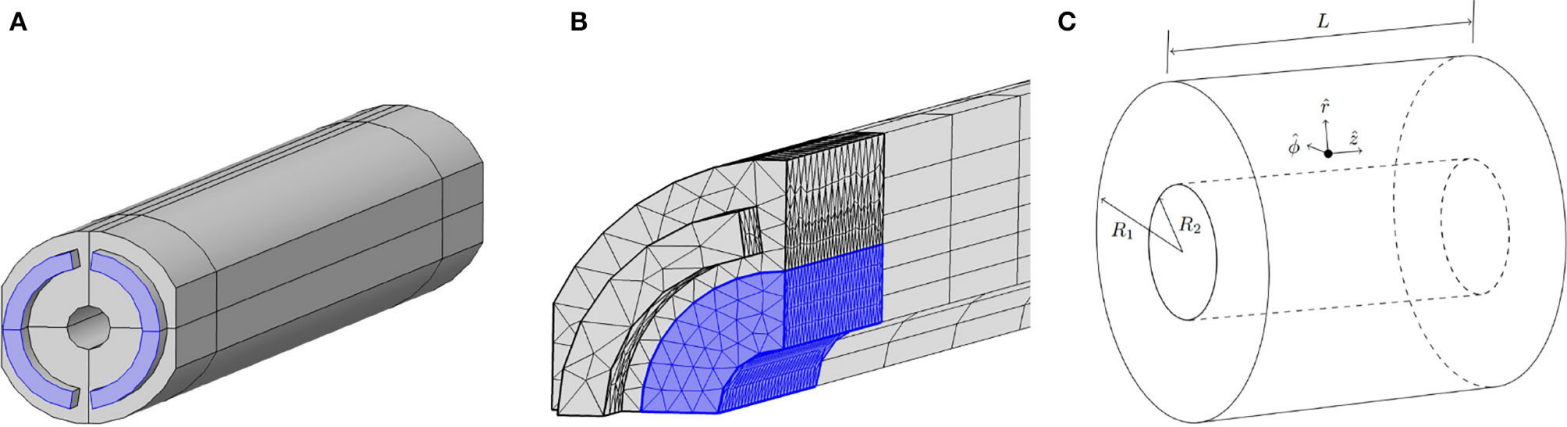
**(A)** Optimal design of the flow channel for the PDFS. The blue semi-annulus shaped regions represent the inflow tubes and the hollow cylinder in the center represents the outside of the fiber. The lines that partition the channel in quarters indicate the symmetry planes of the geometry and show that the channel can be partitioned into four equal parts. The development length that is considered for mesh refinement is indicated with the lines around the outside of the flow channel. Compared to the existing experimental design, shown in Figures 1A,C, all dimensions are decreased: the fiber radius here is 0.25mm, the flow channel radius is 1.1mm, the length of the device is still 2.5cm and the fiber is placed concentrically in the flow channel. Detailed dimensions can be found in the Supplementary Material. **(B)** The mesh that is used to compute the velocity profile numerically in the optimal geometry. Only the first 8mm of the region near the inlet is shown here; the mesh is similar near the outlet. The blue region indicates the domain near the fiber where the mesh is refined. Note that to speed up computation only a quarter of the device is meshed, exploiting the symmetries in the system. To retrieve the full velocity profile in the PDFS, we could then mirror our solution. **(C)** Concentric cylinders with inner radius *R*_2_, outer radius *R*_1_, and length *L*. The unit vectors $\left(ẑ,\hat{r},\hat{\phi }\right)$ in cylindrical coordinates are depicted at a point between the cylinders, where the fluid will flow in the ẑ direction. The inner cylinder represents the outside of the cell layer. This geometry is used to approximate the PDFS; a comparison in terms of shear stress between the two geometries is described in section 3.4.

We have determined the flow in three different geometries. In order of increasing stress homogeneity (and thus potentially a more homogeneous monolayer of differentiated cells) they are: the experimental geometry from Figure 1, the optimal geometry from Figure 2A and concentric cylinders as in Figure 2C. Without any practical constraints, we could achieve a purely homogeneous shear stress upon embedding the fiber in an infinitely long concentric cylinder. The suggested optimal design, identified in Figure 2A, approaches this theoretical optimum as well as possible, with the practical constraints taken into account. Note that the dimensions of the optimal design are decreased compared to that of the experimental design, to allow for relevant shear stresses at a lower flow rate.

### 2.12. Analytic Methods

For the analytic calculations of axial flow between two concentric cylinders as in Figure 2C, we consider a purely pressure driven flow in the longitudinal *z*-direction. Based on the peristaltic pump, we assume the pressure gradient *G* to be varying harmonically with steady offset *G*_0_ and amplitude *G*_1_ = ϵ*G*_0_, thus we have

(3) $$\begin{array}{r} -\frac{\partial p}{\partial z}\equiv G\left(t\right)=G_{0}\left(1+\epsilon \sin\left(\omega t\right)\right), \end{array}$$

where ω is the (angular) driving frequency. We used symmetry arguments and the pressure gradient from Equation (3), to simplify the Stokes equations. Pulsatile flow in a simple tube is well-studied in Leal (2007) and we followed similar (but not identical) calculation steps to find the flow profile between concentric cylinders (see Supplementary Material for detailed calculations). The governing equations were written in adimensional form, to reveal that the functional form of the velocity profile in this system depends on only three dimensionless parameters: the ratio of the cylinders radii *R*_2_/*R*_1_, the relative amplitude ϵ and the Strouhal number $R_{\omega }=\frac{\omega R_{1}^{2}}{\nu }$, which can be viewed as a dimensionless frequency. Here, ν = η/ρ = 7.8·10^−7^m^2/s is the kinematic viscosity.

### 2.13. Numerical Methods

Numerical studies were performed in the experimental and optimal geometry using the software COMSOL Multiphysics® V5.2. We used a 3D model with the creeping-flow module from the single-phase flow package. We used no-slip boundary conditions (BC) on all the walls and on the outside of the fiber, with an incoming Poiseuille mass flux BC at the inlet and a normal-stress-free BC at the outlet. For steady-state computation of the flow in the experimental geometry, a standard tetrahedral mesh was used with a refinement around the fiber for higher accuracy in the shear stress. The element size was determined empirically until a stable solution was found. To speed up time-dependent computations in the optimal geometry, the computational domain was divided into three parts, (i) the vicinity of the fiber, with a fine mesh of tetrahedral basis elements, (ii) the bulk of the device with a swept mesh of hexahedrons as basis elements, and (iii) the vicinity of the inlet and outlet with tetrahedral basis elements. The extent of the domains are determined using the boundary layer of the flow from time-dependent analytical approximations similar to those in Leal (2007) and an analytic result for the development length as described by Durst et al. (2005). We used about 2.5 times the development length to determine the length *L*_*m*_ of region (iii) in the mesh. This resulted in *L*_*m*_≈2.5*l*_*d*_≈1.5*D*_*H*_, with *l*_*d*_ the development length and *D*_*H*_ the hydraulic diameter as written by Langerak (2019). In the optimal geometry, this resulted in *L*_*m*_ = 0.6mm for the in- and outlets and *L*_*m*_ = 3.3mm for the flow channel. We used $\delta =\sqrt{\frac{\nu }{\omega }}$ for the thickness of the boundary layer (Langerak, 2019), and we refined the mesh over a thickness of 3·δ. This resulted in a refined mesh around the fiber with a thickness of 0.33mm. The resulting mesh in the optimal geometry, illustrated in Figure 2B, consists of 34,788 elements. The numerical time-dependent studies were performed for a real time flow of five oscillations and it was confirmed both theoretically (Langerak, 2019) and numerically that this was sufficient for the transient regime to die off.

### 2.14. Parameters for Design Comparison

To compare the numerical calculations in the optimal design (Figure 2A) with the analytic calculations between concentric cylinders (Figure 2C), we used one characteristic set of parameters. For the inner cylinder radius, we have the fiber radius and the cell height (from Hidalgo et al., 1989), which gives *R*_2_ = 250μm+30μm = 0.28mm. The outer radius is minimized, given practical constraints for device strength, and yields *R*_1_ = 1.1mm. This gives a radii ratio of *R*_2_/*R*_1_≈0.2545. We choose experimentally realistic flow rates of *Q*_0_ = 125μL/min for the average flow and *Q*_1_ = *Q*_0_/2 for the amplitude of the oscillatory flow. With a cylinder length of *L* = 2.5cm, this yielded pressure gradients of *G*_0_ = 7.9Pa/m and *G*_1_ = 22.1Pa/m over the concentric cylinder flow channel (see Supplementary Material for this calculation). Thus, we have ϵ = *G*_1_/*G*_0_ = 2.8, and the pressure differences over the flow channel are Δ*p*_0_ = *LG*_0_ = 0.20Pa and Δ*p*_1_ = *LG*_1_ = 0.55Pa. With the chosen peristaltic pump, the average flow rate of *Q*_0_ = 125μL/min can be achieved with a pump setting of about 50 rotations per minute. Given that the pump has 12 rollers, we approximated the oscillation frequency of the pressure gradient as $\frac{\omega }{2\pi }=f=12\cdot 50\text{RPM}=10\text{Hz}$. This yielded for the Strouhal number $R_{\omega }=\omega R_{1}^{2}/\nu \approx 97.3\gg 1$, which indicates that the time-dependent nature of the solution cannot at all be neglected for the final results; we can not approximate our solution with a quasi steady-state.

## 3. Results and Discussion

### 3.1. Characterization of the Flow Around the Hollow Fiber Membrane in the Pump-Driven Flow System

**Figure 3:**
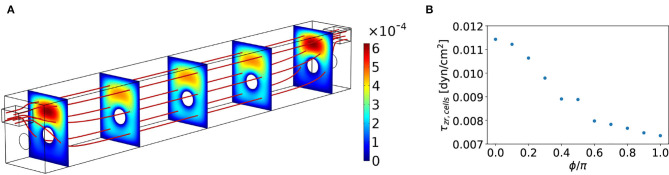
**(A)** Steady-state velocity field *u*_*z*_(**r**) in the experimental flow channel, the magnitude is indicated with the colorbar in (m/s). The red lines indicate streamlines and the cut planes show the velocity magnitude. The dimensions are as indicated in Figure 1C and the channel has a total width of 3 mm and height of 3.65 mm. An aqueous fluid with viscosity η= 0.78mPa s and density ρ= 997.8 kg/m^3^ flows through the device at a steady flow rate of *Q*_0_= 125 μL/min. **(B)** Shear stress on the cells τ_*zr, cells*_ in the center of the microfluidic device as a function of the azimuthal angle ϕ around the fiber. Here, ϕ = 0 corresponds to the top of the fiber.

A numerical evaluation of the steady state flow in the experimental geometry from Figure 1 is performed using COMSOL and depicted in Figure 3A. In the bulk of the channel, we verified that we have (essentially) parallel streamlines, indicating a unidirectional flow; thus the only significant contribution to the flow in the centre of the device is in the longitudinal direction. We confirmed that the in- and outlet effects occur over a length scale of only a few a millimeter, which is negligible for the experiment.

Given the laminar velocity profile *u*_*z*_(*r*, ϕ) in the bulk of the device, we can evaluate the shear stress $\tau_{zr}\left(r,\phi \right)=\eta \frac{\partial u_{z}\left(r,\phi \right)}{\partial r}$ numerically. Here, *r* is still the radial distance from the center of the fiber and ϕ is the azimuthal angle, defined with ϕ = 0 on the top of the fiber. Note that we are most interested in the shear stress on the cells, given by

(4) $$\begin{array}{r} \tau_{zr,cells}(\phi )=\eta {\frac{\partial u_{z}(r,\phi )}{\partial r}|}_{r=R_{2}}. \end{array}$$

The numerical evaluation of the shear stress on the cells around the fiber is depicted in Figure 3B. With a steady flow rate of *Q*_0_ = 125μL/min, we observed a heterogeneous shear stress on the cells around the fiber with a maximum of about 11.5·10^−3^dyne/cm^2 at the top of the fiber and a minimum of about 7.4·10^−3^dyne/cm^2 at the bottom. Thus, a relative deviation from the average of 22%. Despite the stress heterogeneity, we did observe that our inflow of *Q*_0_ = 125μL/min gives a shear stress that is of the order of magnitude of the required physiological shear stress for the intestinal cells to differentiate (as determined by Olesen et al., 1988; Lentle and Janssen, 2008; Ishikawa et al., 2011). Our measurements on cell differentiation were performed in this device for flow rates around this value. A more detailed analysis of a more homogeneous shear stress on the cells in the optimal geometry is described after the experimental results.

### 3.2. Experimental Results

An overarching goal of this work was to develop a gut-on-a-chip model that can be combined with other organs, primarily kidney, to study the effect of uremic toxins in chronic kidney disease on the gut. For that purpose, p-cresol was chosen as a model compound for uremic toxins.

**Figure 4:**
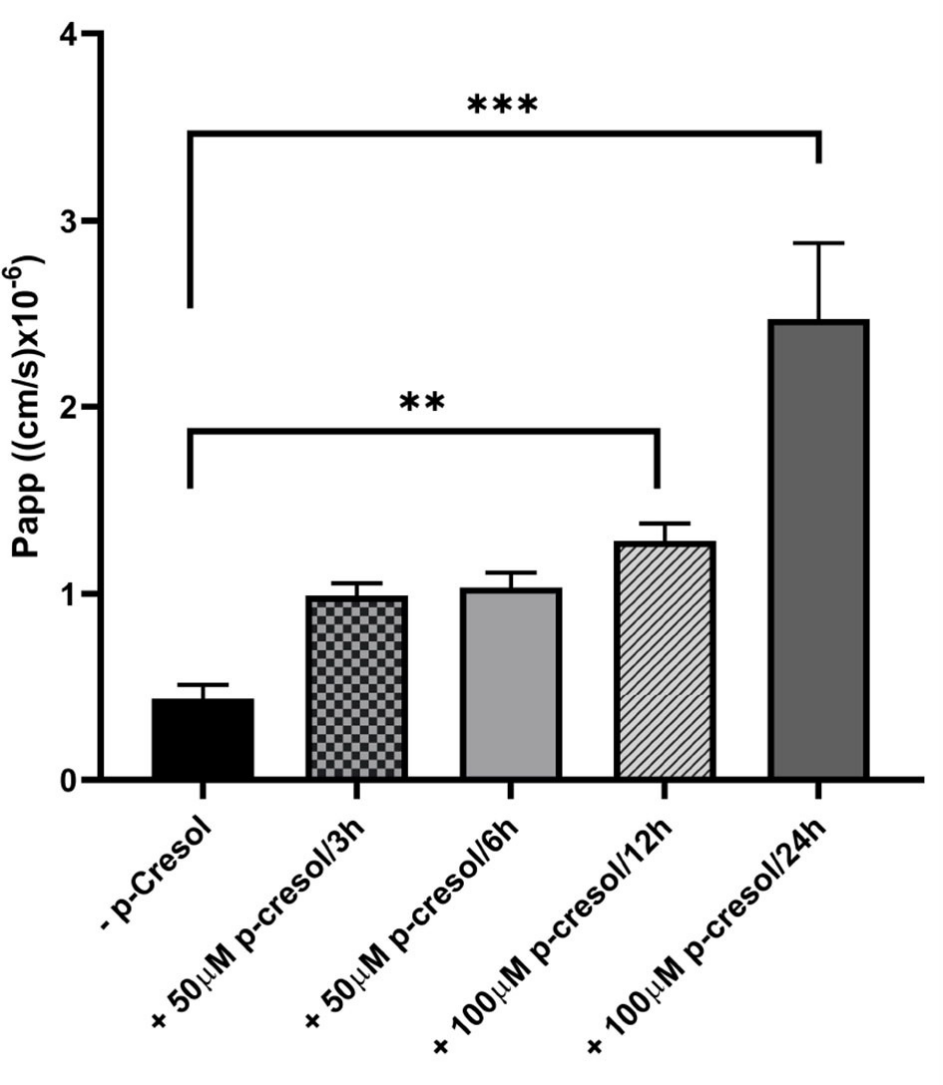
Monolayer integrity of Caco-2 cells in GDFS cultures when treated with or without p-cresol at 50 or 100 μM for 3, 6, 12, or 24 h. Data are shown as mean ± SD, *n* = 4. **p < 0.01; ***p < 0.001.

To determine the optimal conditions and establish a baseline, Caco-2 cells were cultured on collagen-coated hollow fibers in the gravity-driven flow system (GDFS) for 3 weeks. L-Dopa/collagen-coated hollow fibers have been previously shown by Chevtchik et al. (2016) not to present any significant mass transfer limitations.

p-Cresol was subsequently added to investigate its effect on monolayer integrity and cell viability, as determined by apparent paracellular permeability (*P*_*app*_) and LDH release, respectively. Exposure to p-cresol resulted in strongly compromised barrier function (Figure 4), due to the toxic nature of this compound and its metabolites on the cells (Vaziri et al., 2015; Gryp et al., 2017).

**Figure 5:**
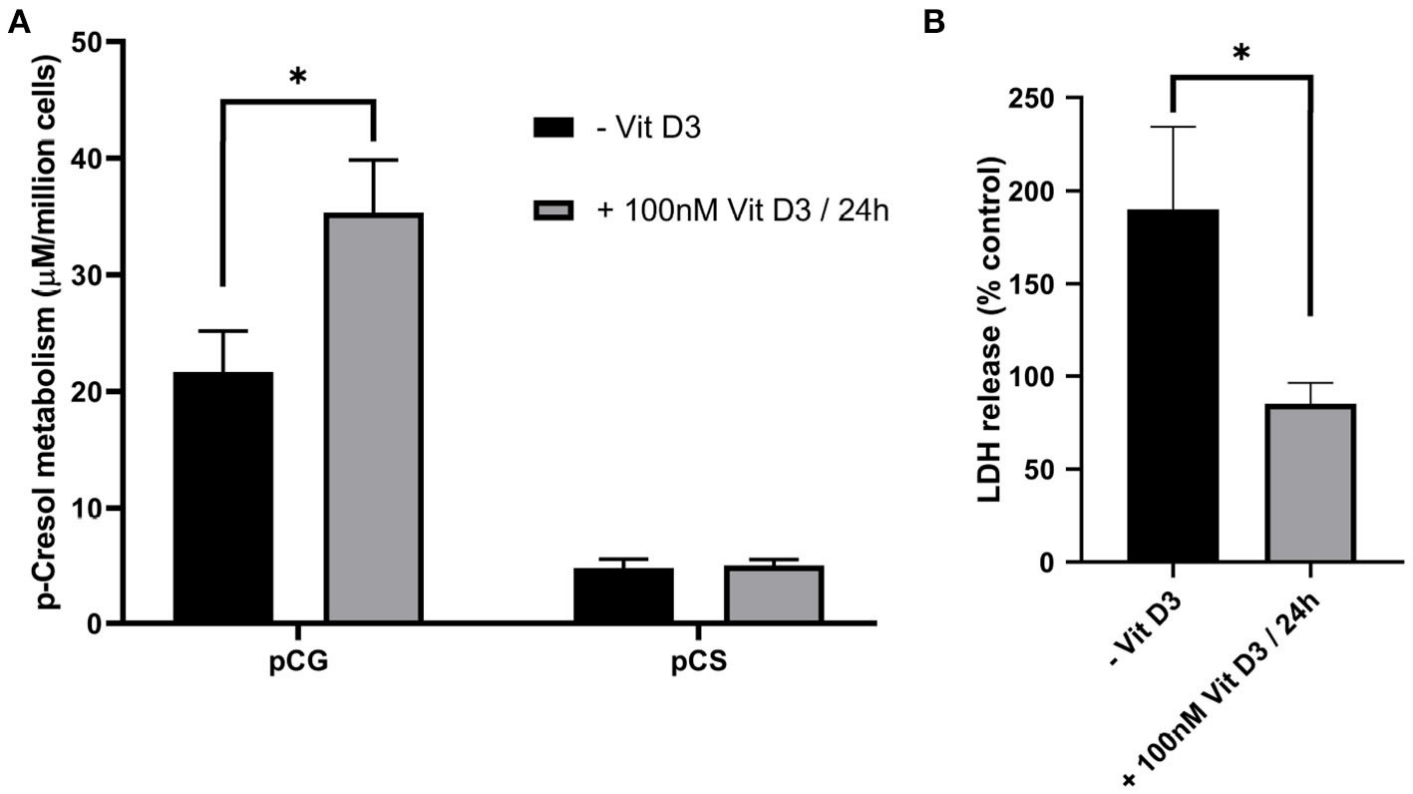
The effect of vitamin D3 pre-treatment on Caco-2 metabolic capacity **(A)** and membrane integrity **(B)** upon subsequent treatment with 50 μM p-cresol for 3 h. Data are shown as mean ± SD, *n* = 4. *p < 0.05.

Vitamin D3 has been shown to improve CYP3A4 induction in Caco-2 cells by Kasendra et al. (2020) and improve tight junction conductance (Chirayath et al., 1998) possibly through the interaction with Claudin-2 gene as shown by Zhang et al. (2015). We found that vitamin D3 treatment prior to p-cresol exposure improved membrane integrity, while enhancing p-cresol metabolism (Figure 5).

**Figure 6:**
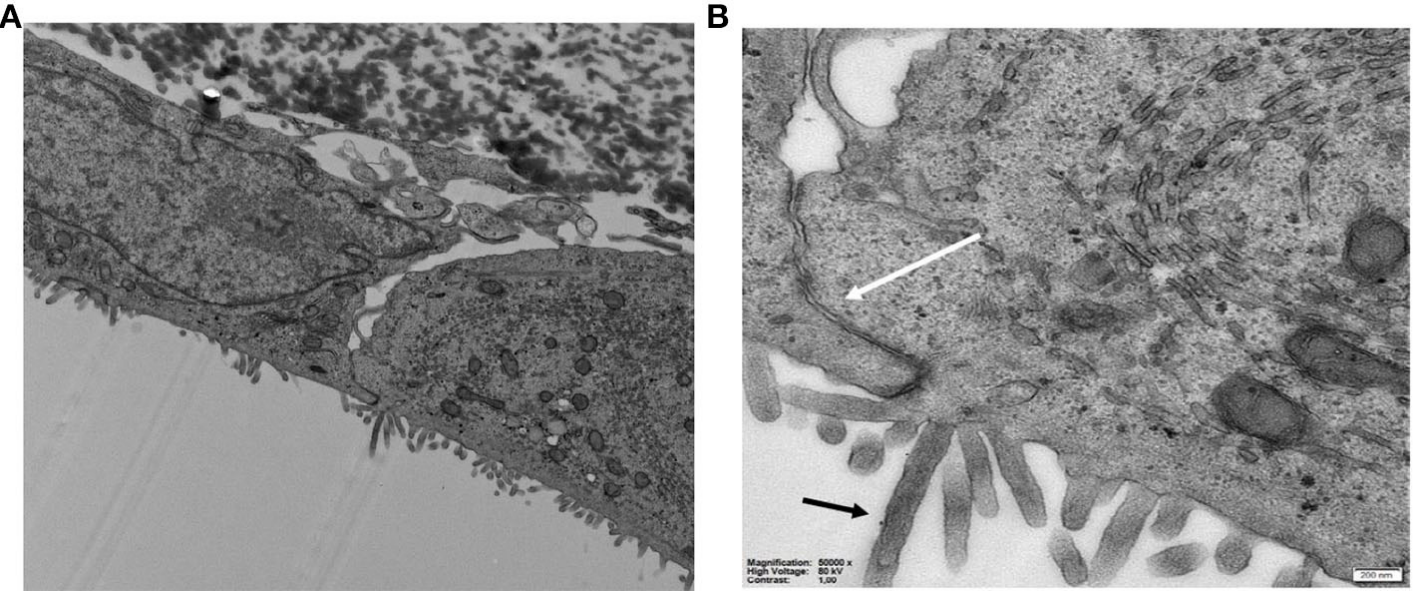
**(A,B)** TEM images of Caco-2 cells on HFM in the GDFS cultures showing **(B)** the tight junctions (white arrow) and microvilli (black arrow).

To verify the differentiation and polarization, cells were examined with TEM and the images obtained (Figure 6) clearly shows the formation of microvilli on the apical membrane and tight-junctions at the lateral side.

Next, the optimization for the pump-driven flow system (PDFS) was performed. We carried out computations to determine the range of flow rates that would result in physiologically relevant shear stresses for gut tubules. Our fluid dynamics' simulation showed a fairly uniform shear stress along almost the whole length of the fiber with the top surface being exposed to the maximal stress (Figure 3). Flow rates of 30, 60, 90, and 125 μL/min, corresponding to maximum shear stresses of 0.003, 0.005, 0.008 and 0.011 dyne/cm^2, were chosen in the physiologically relevant range.

GDFS has been shown to improve Caco-2 differentiation and functionality compared to static cultures (Jochems et al., 2019). Similarly, shear stress has been shown to improve Caco-2 differentiation compared to static cultures (Kim and Ingber, 2013; Shim et al., 2017). Additionally, a time-course experiment investigating the effect of shear stress on *P*_*app*_ has been recently reported by Kasendra et al. (2020). In this work, *P*_*app*_ of Duodenum-on-a-chip was shown to decrease over time for cells cultured under shear stress.

**Figure 7:**
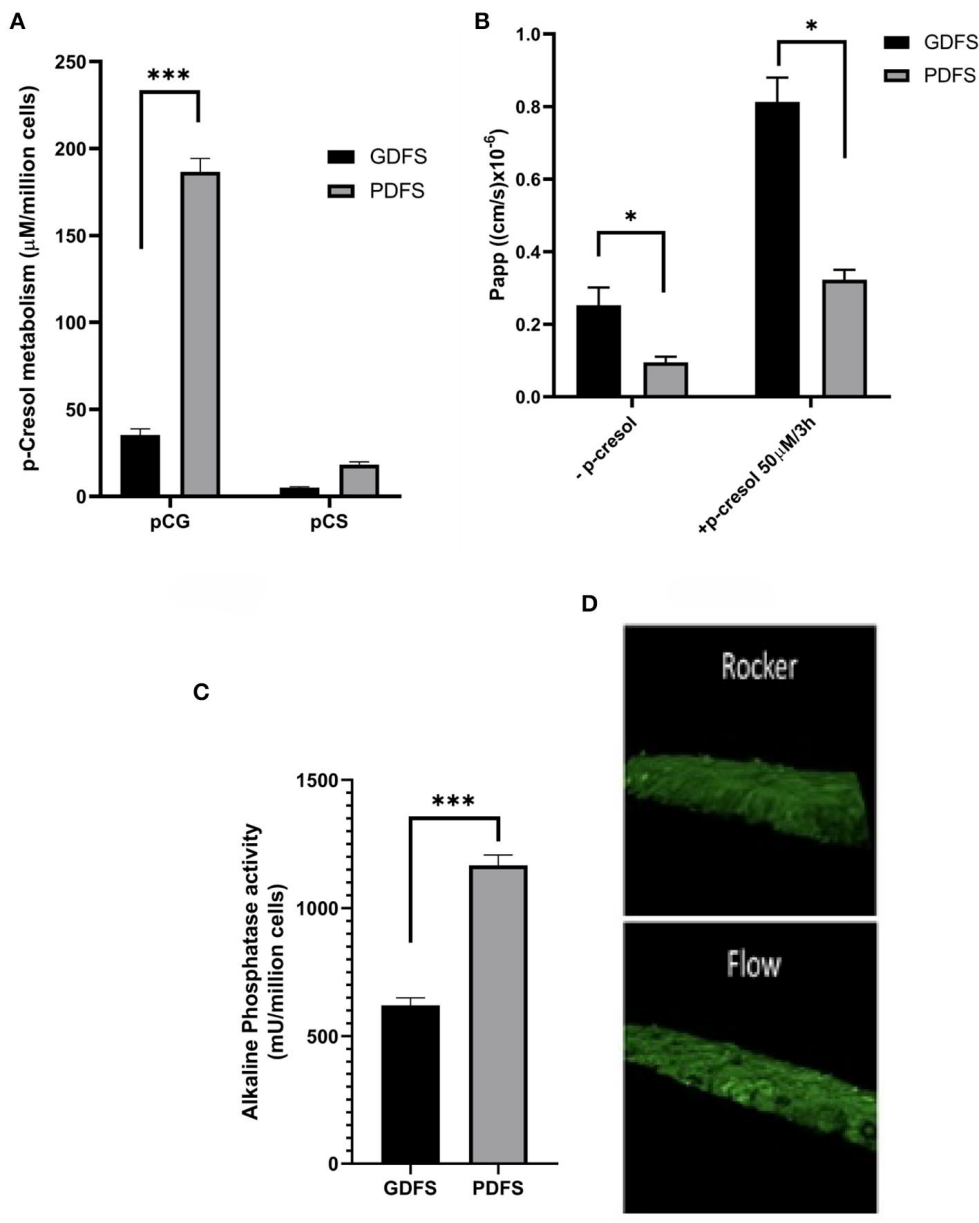
Functional analysis of Caco-2 cells in GDFS vs. PDFS. PDFS exhibited higher p-cresol metabolic capacity into p-cresol glucuronide (pCG) and p-cresol sulfate (pCS) **(A)**, tighter monolayer formation **(B)**, improved brush border activity **(C)** and better differentiation in terms of villi formation **(D)**. Data are shown as mean ± SD, *n* = 2–4. *p < 0.05; ***p < 0.001.

In our work, Caco-2 cells cultured in a GDFS for 21 days were compared to ones cultured in a PDFS for 10 days. We have shown that uni-directional flow achieved by the PDFS was crucial for the formation of a tight monolayer as evident by the apparent permeability (*P*_*app*_) (Figure 7B) when compared to the bi-directional flow of the GDFS.

Moreover, Caco-2 cells in the PDFS formed villi and crypts reminiscent of intestinal villi (Figure 7D). The protective and metabolism-inducing effect of Vit. D3 on Caco-2 cells was augmented in the PDFS as compared to the GDFS (Figures 7A,B). Additionally, the brush-border activity expressed in terms of alkaline phosphatase activity was significantly improved under uni-directional flow in the PDFS compared to the bi-directional flow of the GDFS (Figure 7C).

**Figure 8:**
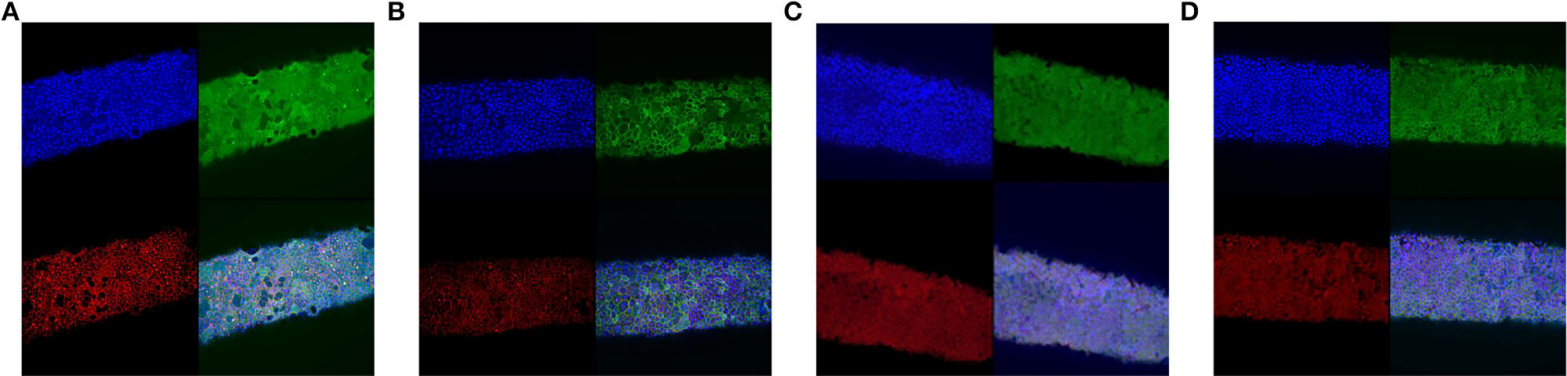
Confocal microscope images of Caco-2 cells in PDFS after 10 days under 30 **(A)**, 60 **(B)**, 90 **(C)**, and 125 **(D)** μL/min. Images show staining for tight-junctions (ZO-1, red), villi (villin, green), and nuclei (DAPI, blue).

Fibers were subjected to these flows for 10 days and analyzed by confocal imaging, showing the formation of tight-junctions without considerable interruptions at 90 and 125 μL/min (Figure 8).

### 3.3. Axial Flow Between Concentric Cylinders

Recall that the flow in our experiment was driven by a peristaltic pump to mimic the time oscillating nature of fluid flow in human organs. In the experimental geometry, we have only determined the steady state flow rate, since the design results in a computationally heavy numerical evaluation. We have determined the full time-harmonic flow rate between concentric cylinders to have a more complete prediction of the shear stress on the cells in our (optimal) experiment.

Solving Equation (2) for axial flow between concentric cylinders and using the spatial derivative from Equation (4), resulted in an explicit expression for the shear stress on the cells of the form (see Supplementary Material for details on the calculation):

(5) $$\begin{array}{r} \tau_{\text{zr,cells}}=G_{0}R_{1}\left(\frac{-R_{2}}{2R_{1}}+\frac{\frac{R_{1}}{R_{2}}-\frac{R_{2}}{R_{1}}}{4\ln\left(\frac{R_{1}}{R_{2}}\right)}\right)\left[1+\epsilon A_{\tau }\sin\left(\omega t+\phi_{\tau }\right)\right]. \end{array}$$

**Figure 9:**
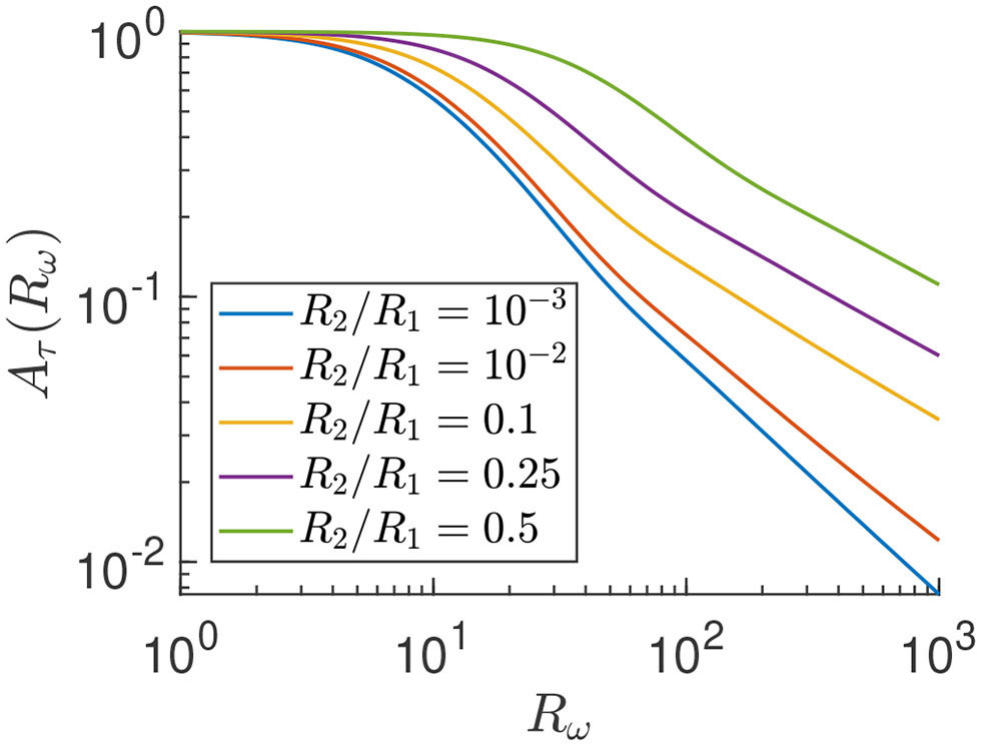
Reduction factor *A*_τ_ of the time-dependent shear stress amplitude compared to the stationary stress as in Equation (5) on log-log scale.

Equation (5) reveals not only a phase shift, ϕ_τ_, between the applied pressure and experienced stress – which is not so relevant given the long duration of exposure to stress – but also a reduction factor *A*_τ_(*R*_ω_, *R*_2_/*R*_1_) of the time-dependent stress amplitude compared to the stationary stress. Explicit expressions of *A*_τ_ and ϕ_τ_ in terms of *R*_ω_ and *R*_2_/*R*_1_ are given in the Supplementary Material. Thus, with Equation (5), we have an explicit evaluation of the shear stress on the cells for all system parameters, which allows for efficient prediction of the shear under any conditions. In Figure 9, we plot *A*_τ_ as a function of *R*_ω_ for a few cylinder geometries *R*_2_/*R*_1_.

In Figure 9 we observe that, in the static limit (*R*_ω_ → 0), there is no reduction of the shear stress, i.e., *A*_τ_ → 1 for each radii ratio. We also confirmed in Langerak (2019) that ϕ_τ_ → 0 in the same *R*_ω_ → 0 limit, such that the shear stress (Equation 4) reduces to a stationary shear stress in the static limit, as predicted. Furthermore, in Figure 9 we see that *A*_τ_ goes to zero algebraically for high frequencies as $R_{\omega }^{-\alpha }$ with a non-universal (radii ratio dependent) exponent α>0 that we do not study in any detail here. Thus, qualitatively, Figure 9 shows that there is a reduction factor *A*_τ_ ≤ 1 of the time-dependent stress compared to the stationary stress, that decreases for increasing driving frequency.

Note that for our parameters, *R*_ω_≈100 and *R*_2_/*R*_1_≈0.25, we have a reduction factor of *A*_τ_≈0.2. This indicates that the time oscillating behavior of the system is non-negligible in terms of the effect on the shear stress and we indeed need the full time-dependent calculations instead of a much easier quasi steady-state approach.

### 3.4. Asymmetry in Optimal Geometry

**Figure 10:**
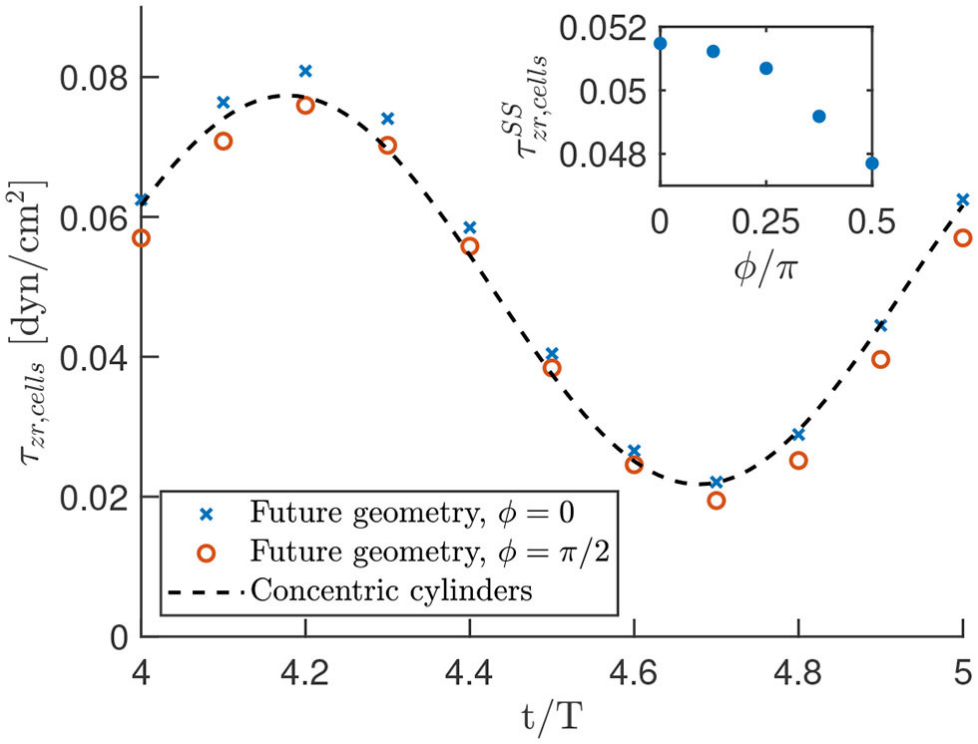
Oscillating shear stress on the cells in the optimal geometry as a function of time. The data points result from the numerical calculations in COMSOL, while the black dashed line is the analytic prediction in a concentric cylinder geometry. These results are obtained with the parameter settings as described in the section 2 and oscillation period *T* = 0.1s. The shown shear stress is the sum of a steady and a time-harmonic contribution. The inset highlights the steady state shear stress on the cells for various values of ϕ/π to indicate the homogeneity of the shear stress.

To use our analytic results for efficient predictions in the optimal geometry, we analyzed the difference in shear stress on the cells in the optimal geometry (Figure 2A) and the concentric cylinder geometry Figure 2C for one set of parameter values as described in the section 2. The analysis is performed for both the steady state and time-oscillating solution, where we used data of the shear stress on points of the fiber for fixed angles ϕ/π = 0, 1/8, 1/4, 3/8, 1/2 in the center of the device. The resulting shear stress on the cells, as well as the analytic prediction is shown in Figure 10.

First, Figure 10, suggests that there is only a small error when we approximate the optimal geometry with concentric cylinders. An analysis of the results yielded a maximum deviation of about 10% in the shear stress between the two geometries. The minimum error was achieved using our analytic results with an outer cylinder of 98.6% of the radius we used to construct the optimal geometry. This suggests that we can use our analytic results as a reasonable approximation of the shear stress in the experiment.

Although the time dependent oscillatory stress is only shown for ϕ/π = 0, 1/2 in the main graph of Figure 10, we verified that the shear stress is in between these values for other azimuthal angles. Furthermore, we see in the inset of Figure 10 that we have a very homogeneous steady-state shear stress in the optimal geometry, with a relative deviation from the average of only 4%. Thus, we observed a nearly homogeneous shear stress on the cells in the optimal geometry, which is well-approximated by our analytic calculations.

## 4. Conclusions

A new intestinal chip was designed using numerical computation of the fluid flow and the result is shown in Figure 1. This three dimensional setup is constructed using an inert biocompatible polylactic acid instead of the common microfabrication with PDMS to minimize solute absorption. The setup does still allow for high-resolution, real-time imaging of biochemical activities. Furthermore, cells are seeded on a hollow fiber, which adds curvature to the cell structure and does not hinder transport of physiological solutes.

With our design, we aimed to optimize cell differentiation toward a tight mono-layer of functional organ cells. With numerical and analytic calculations, we found a design that result in a tuneable, time oscillating and (nearly) homogeneous shear stress on the cells in the physiologically relevant regime.

The cells in our flow setup exhibited a higher p-cresol metabolic capacity, tighter monolayer formation, improved brush border activity (alkaline phosphatase) and better differentiation in terms of villi formation (Figure 7) compared to previous studies like Jochems et al. (2019). Thus, this novel microphysiological system is already a promising tool for studies of the metabolic functions of the intestine.

Furthermore, we have shown that we can improve the homogeneity of the shear stress even further with a design similar to concentric cylinders (Figure 2A). Using analytic calculations, we can fully predict and tune the time-dependent shear stress on the cells with an error of order 10%. We predicted a nearly homogeneous shear stress on the cells with a relative deviation of 4% around the fiber, compared to a 22% deviation in the experimental geometry. Since the shear stress is determined as a function of all free system parameters, in future studies, these analytic results can be used to efficiently predict the stress for all pressure gradient ratios ϵ and driving frequencies ω.

In addition to design optimization to ensure homogenous shear stress, future work will also focus on optimization of the biological component through using organoids instead of Caco-2 cells; thereby recapitulating the complexity of the intestinal tissue. Furthermore, the use of Caco-2 is not ideal since the use of organoids or iPSC-derived cells will better recapitulate the complexity of the *in vivo* intestinal tissue (Dutton et al., 2019). However, this was a first step to establish a novel gut-on-a-chip and in the future more experiments optimizing such a chip both in terms of fluid dynamics and biological relevance to the intestine will be carried out.

In conclusion, we have shown that our new intestinal chip gives an improved functional mono-layer of cells. Finally, it has the potential to study other organs (in a modular way) at a later stage, since the shear stress is fully tuneable.

## Data Availability Statement

Numerical datasets generated for this study are included in the article/Supplementary Material. All experimental data are available on request.

## Author Contributions

NL performed the numerical and analytic calculations under supervision of RR. HA performed the experiments in collaboration with IM and HE under supervision of RM. YL constructed the 3D microfluidic device under supervision of JM. NL, HA, and RM drafted this manuscript. All authors provided critical feedback and helped shape the results analysis and manuscript.

## Conflict of Interest

The authors declare that the research was conducted in the absence of any commercial or financial relationships that could be construed as a potential conflict of interest.
