# Supplementary material for: A Theoretical and Experimental Study to Optimize Cell Differentiation in a Novel Intestinal Chip
Source: Front Bioeng Biotechnol. 2020 Jul 24;8:763. doi: 10.3389/fbioe.2020.00763 (PMC7393935; doi:10.3389/fbioe.2020.00763)
Supplement: Supplementary file 1 [file Data_Sheet_1.PDF]

Supplementary Material

Nomenclature

$\bar{x}$	Adimensional form of quantity x
δ	Thickness of the boundary layer for time oscillating flow
ϵ	Relative pressure gradient, $\epsilon = G_1/G_0$
η	Dynamic viscosity
ν	Kinematic viscosity
ω	Angular driving frequency of the oscillating pressure gradient
ϕ_τ	Phase shift between applied pressure gradient and shear stress on the cells
ρ	Mass density
τ	Shear stress
A_τ	Reduction factor of time-dependent shear stress amplitude compared to stationary stress on the cells
D_H	Hydraulic diameter
$G_{0,1}$	Steady offset (0) and amplitude (1) of the time-harmonic pressure gradient
L	Length of the flow channel and fiber
l_d	Development length
L_m	Length of the region in the optimal geometry where the mesh is refined
p	Pressure
$Q_{0,1}$	Steady offset (0) and amplitude (1) of the time-harmonic flow rate
R_ω	Strouhal number, in our concentric cylinder geometry defined as $R_\omega = \omega R_1^2/\nu$
$R_{1,2}$	Outer (1) and inner (2) radius of a concentric cylinder geometry
T	Oscillation period
t	Time
u	Fluid velocity

Axial flow between concentric cylinders

As described in the main text, we computed the axial flow between concentric cylinders with inner radius R_2, outer radius R_1 and length L. We restricted attention to the low-Reynolds number regime, where the flow is laminar, without any turbulence. The governing Stokes equations

$$\rho \frac{\partial \mathbf{u}}{\partial t} = -\nabla p + \eta \nabla^2 \mathbf{u}, \quad \nabla \cdot \mathbf{u} = 0, \tag{1}$$

were solved for the velocity profile $\mathbf{u}(\mathbf{r}, t)$ and the pressure $p(\mathbf{r}, t)$ at position $\mathbf{r}$ and time t. We used no-slip boundary conditions on the inner- and outer cylinder wall. We simplified these equations using geometrical properties of our system.

In our systems, the length of the cylinders L is much larger than the radius R_1, that is $L/R_1 \gg 1$, such that we can neglect inlet and outlet effects of the flow. So, away from the in- and outlet, we have an approximate translational symmetry in the axial direction. This symmetry combined with our assumption of a laminar flow implies there will only be a nonzero contribution to the velocity field in the longitudinal direction such that $\mathbf{u}(\mathbf{r}, t) = (0, 0, u_z(\mathbf{r}, t))$. Also, we have rotational symmetry in this geometry, so in cylindrical coordinates we can write $u_z(r, \phi, z, t) = u_z(r, z, t)$. Then, with the incompressibility condition, $\nabla \cdot \mathbf{u} = 0$, we see that u_z is independent of z, so we have $u_z = u_z(r, t)$. Since the r and ϕ component of $\mathbf{u}$

are zero, it follows from the Stokes equations (1) that

$$\frac{\partial p}{\partial r} = \frac{\partial p}{\partial \phi} = 0. \tag{2}$$

So the pressure is at most a function of z and t only, $p = p(z,t)$. By using the geometrical properties and the translational invariance along the z-direction, we have reduced the vector equations (1) to the scalar equations:

$$\begin{cases} \rho \dfrac{\partial u_z(r,t)}{\partial t} = \eta \dfrac{1}{r}\dfrac{\partial}{\partial r}\left(r \dfrac{\partial u_z(r,t)}{\partial \bar{r}} \right) - \dfrac{\partial p(z,t)}{\partial z} \\ u_z(r = R_1, t) = 0; \\ u_z(r = R_2, t) = 0; \\ u_z(r, t = 0) = 0. \end{cases} \tag{3}$$

When we take the spatial derivative $\frac{\partial}{\partial z}$ of the Stokes equation (3), we observe that $\frac{\partial^2 p}{\partial z^2} = 0$ and thus our pressure gradient $\frac{\partial p}{\partial z}$ must be independent of z. Based on the peristaltic pump, we assume the pressure gradient G to be varying harmonically with steady offset G_0 and amplitude $G_1 = \epsilon G_0$ in the longitudinal z-direction. Thus we have

$$-\frac{\partial p}{\partial z} \equiv G(t) = G_0(1 + \epsilon \sin(\omega t)), \tag{4}$$

where ω is the (angular) driving frequency.

To gain insight in the nature of the flow and the parameters it depends on, we nondimensionalize our equations (3) using

$$\bar{u}_z = \frac{u_z}{G_0 R_1^2/\eta}, \quad \bar{r} = \frac{r}{R_1}, \quad \hat{t} = \frac{t}{R_1^2/\nu}. \tag{5}$$

This results in:

$$\begin{cases} \dfrac{\partial \bar{u}_z(\bar{r},\hat{t})}{\partial \hat{t}} = \dfrac{1}{\bar{r}}\dfrac{\partial}{\partial \bar{r}}\left(\bar{r} \dfrac{\partial \bar{u}_z(\bar{r},\hat{t})}{\partial \bar{r}} \right) + 1 + \epsilon \sin\left(R_\omega \hat{t}\right)); \\ \bar{u}_z(\bar{r} = 1, \hat{t}) = 0; \\ \bar{u}_z(\bar{r} = R_2/R_1, \hat{t}) = 0; \\ \bar{u}_z(\bar{r}, \hat{t} = 0) = 0, \end{cases} \tag{6}$$

where we defined R_ω as

$$R_\omega = \frac{\omega R_1^2}{\nu}. \tag{7}$$

We can see by inspection of (6) that the nature of the flow depends on 3 dimensionless parameters only: R_2/R_1, ϵ and the Strouhal number R_ω. This tells us that the functional form of the solution depends *only* on these ratios of radii, pressure gradients and time scales. The solution will of course still scale with the characteristics we that we defined in equations (5).

Because of the linearity of equations (6) we can solve for the velocity field in two parts:

$$\bar{u}_z = \bar{u}_z^{(0)} + \epsilon \bar{u}_z^{(1)}. \tag{8}$$

Here, $\bar{u}_z^{(0)}$ and $\epsilon \bar{u}_z^{(1)}$ represent the flow profile due to the stationary pressure gradient G_0, and to the oscillatory part $G_0 \epsilon \sin(\omega t)$ respectively.

In the thesis related to this work, we have shown explicitly[1] that the transient start-up regime of the flow dies of exponentially, similar to that in a simple tube[2]. This dimensional timescale is of the order of seconds, and thus irrelevant for the experimental timescales of days and we will neglect it in this work.

Using standard methods, after asymptotically large times, we find the steady state velocity profile as

$$\bar{u}_z^{(0)}(\bar{r}) = \frac{1}{4}\left[1 - \bar{r}^2 - \frac{1 - (R_2/R_1)^2}{\ln(R_1/R_2)} \ln\left(\frac{1}{\bar{r}}\right) \right]. \tag{9}$$

To solve for the time oscillating part of the solution $\bar{u}_z^{(1)}$, we make the Ansatz that the velocity will also be oscillating with the dimensionless frequency R_ω and arbitrary phase difference with the pressure gradient. Thus, we write after asymptotically large times:

$$\bar{u}_z^{(1)} = \mathrm{Im}\left(e^{iR_\omega \hat{t}} H(\bar{r})\right), \tag{10}$$

where $H(\bar{r})$ is a spatially dependent function that incorporates the phase difference. When we substitute this Ansatz into the harmonic part of (6), we arrive at

$$\begin{cases} \dfrac{\partial^2 H(\bar{r})}{\partial \bar{r}^2} + \dfrac{1}{\bar{r}}\dfrac{\partial H(\bar{r})}{\partial \bar{r}} - iH(\bar{r})R_\omega = -1; \\ H(\bar{r}=1) = 0; \\ H(\bar{r} = R_2/R_1) = 0. \end{cases} \tag{11}$$

This linear non-homogeneous ordinary differential equation can be solved using standard methods, to separate the solution in a particular and homogeneous solution. In the resulting homogeneous equation, we can recognize a 0th order Bessel equation. Solving these equations, results in:

$$H(\bar{r}) = \frac{i}{R_\omega}\left[-1 + AJ_0\left(\sqrt{\frac{R_\omega}{i}}\bar{r}\right) + BY_0\left(\sqrt{\frac{R_\omega}{i}}\bar{r}\right)\right], \tag{12}$$

with J_0, Y_0 the Bessel functions of the first and second kind and A, B two integration constants. They are computed as:

$$\begin{cases} A = \dfrac{Y_0\left(\sqrt{\frac{R_\omega}{i}}\frac{R_2}{R_1}\right) - Y_0\left(\sqrt{\frac{R_\omega}{i}}\right)}{J_0\left(\sqrt{\frac{R_\omega}{i}}\right)Y_0\left(\sqrt{\frac{R_\omega}{i}}\frac{R_2}{R_1}\right) - Y_0\left(\sqrt{\frac{R_\omega}{i}}\right)J_0\left(\sqrt{\frac{R_\omega}{i}}\frac{R_2}{R_1}\right)}; \\ B = \dfrac{J_0\left(\sqrt{\frac{R_\omega}{i}}\right) - J_0\left(\sqrt{\frac{R_\omega}{i}}\frac{R_2}{R_1}\right)}{J_0\left(\sqrt{\frac{R_\omega}{i}}\right)Y_0\left(\sqrt{\frac{R_\omega}{i}}\frac{R_2}{R_1}\right) - Y_0\left(\sqrt{\frac{R_\omega}{i}}\right)J_0\left(\sqrt{\frac{R_\omega}{i}}\frac{R_2}{R_1}\right)}. \end{cases} \tag{13}$$

This gives us the time oscillating part of the solution $\bar{u}_z^{(1)}$ upon inserting our result of $H(r)$ in equation (10). For completeness, a visual representation of the oscillation velocity field is presented in Fig. 1. Here we see that we have a spatially dependent phase difference for high Strouhal number which results in a nontrivial velocity field. The full solution of the velocity field $u_z(r,t)$ is found by combing equations (5), (8), (9), (10) and (12). We will not write this explicitly, but instead focus our attention to the shear stress on the cells.

Shear stress on the cells

Recall that because we solve in a rotationally symmetric and (approximatetly) infinitely long geometry, we find a homogeneous shear stress on the cells which is non-zero in the z-direction only. Upon evalutating $\tau_{zr,cells}(\phi) = \eta \left.\frac{\partial u_z(r,\phi)}{\partial r}\right|_{r=R_2}$, the expression takes the dimensional form

$$\tau_{\text{zr,cells}} = G_0 R_1\left(\frac{-R_2}{2R_1} + \frac{\frac{R_1}{R_2} - \frac{R_2}{R_1}}{4\ln\left(\frac{R_1}{R_2}\right)}\right)\left[1 + \epsilon A_\tau \sin\left(\omega t + \phi_\tau\right)\right], \tag{14}$$

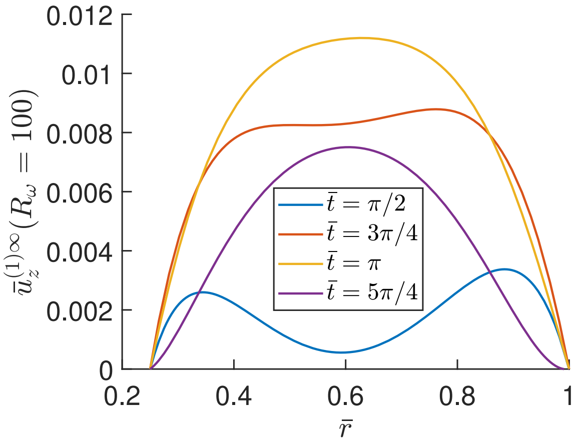

Figure 1: Harmonic velocity field during a quarter of the oscillation period for high Strouhal number $R_\omega = 100$. These graphs were generated using $R_2/R_1 = 1/4$, similar to the radii ratio in the optimal geometry. The superscript ∞ indicates that this is the velocity field after asymptotically large times.

where A_τ, ϕ_τ are the amplitude and phase of the shear stress due to the oscillatory pressure gradient. They are evaluated as:

$$\begin{cases} A_\tau = \dfrac{-\mathrm{Im}(f)}{\sin(\phi_\tau)} = \dfrac{-\mathrm{Re}(f)}{\cos(\phi_\tau)}; \\ \phi_\tau = \tan^{-1}\left(\dfrac{\mathrm{Im}(f)}{\mathrm{Re}(f)}\right); \\ f(R_\omega) = \sqrt{\dfrac{i}{R_\omega}}\left[AJ_1\left(\sqrt{\dfrac{R_\omega}{i}}\dfrac{R_2}{R_1}\right) + BY_1\left(\sqrt{\dfrac{R_\omega}{i}}\dfrac{R_2}{R_1}\right)\right]. \end{cases} \tag{15}$$

Here, J_1, Y_1 are first order Bessel functions of the first and second kind respectively. An analysis of this result is described in the main text.

Relation between flow rate and pressure gradient

Recall that in our analytic calculations, we used the pressure gradient G as the driving force for the fluid flow. On the other hand, in the experiment, a flow rate Q is imposed on the microphysiological system due to the peristaltic pump. Here, we relate these two quantities, G and Q, such that we can compare the theoretical and experimental results.

Let us define the flow rate (again after asymptotically large times) as a steady and harmonic contribution, similar to our pressure gradient (4);

$$Q = Q_0 + Q_1 \sin(\omega t + \phi_Q), \tag{16}$$

with Q_0, Q_1 the amplitudes of the steady and harmonic flow rates and ϕ_Q the phase difference with the pressure gradient. Note that we already anticipated on a harmonic flow rate after long times, since we also found this for the velocity profile. By definition, the flow rate Q is the volume of fluid which passes the system per unit time and is evaluated in cylindrical coordinates as

$$Q(t) \equiv \int_{\phi=0}^{2\pi}\int_{r=R_2}^{R_1} \mathrm{d}r\mathrm{d}\phi \;\, r u_z(r,t). \tag{17}$$

We evaluated this integral given the velocity field we found between concentric cylinders[1] and upon lengthy algebra we found relations between the flow rate and pressure gradient. For the steady state flow rate, we find

$$Q_0 = G_0 \frac{A_{\text{flow}}}{8\eta}\left[R_1^2\left(1 - \frac{1}{\ln(R_1/R_2)}\right) + R_2^2\left(1 + \frac{1}{\ln(R_1/R_2)}\right)\right], \tag{18}$$

where $A_{\rm flow} = \pi(R_1^2 - R_2^2)$ is the area through which the fluid flow.

For the time oscillating amplitude Q_1, we find

$$Q_1 = G_1 \frac{R_1^4}{\eta} A_Q, \tag{19}$$

where A_Q is a reduction factor, depending on the driving frequency ω and radii ratio R_2/R_1. Its explicit expression is rather lengthy and of a similar form as our results for the reduction factor A_τ for the shear stress;

$$\begin{cases} A_Q = \dfrac{{\rm Im} f_Q}{\sin(\phi_Q)} = \dfrac{{\rm Re} f_Q}{\cos(\phi_Q)}, \\ \phi_Q = \tan^{-1}\left(\dfrac{{\rm Im} f_Q}{{\rm Re} f_Q}\right), \\ f_Q = \dfrac{2\pi i}{R_\omega}\left\{\dfrac{1}{2}\left(\dfrac{R_2^2}{R_1^2} - 1\right) + \dfrac{1}{R^*}\left[A\left(J_1(R^*) - \dfrac{R_2}{R_1} J_1\left(R^* \dfrac{R_2}{R_1}\right)\right) + \right.\right. \\ \qquad\qquad \left.\left. + B\left(Y_1(R^*) - \dfrac{R_2}{R_1} Y_1\left(R^* \dfrac{R_2}{R_1}\right)\right)\right]\right\}, \\ R^* = \sqrt{\dfrac{R_\omega}{i}}. \end{cases} \tag{20}$$

Thus, we have found the relations between the flow rate and the pressure gradient, which allows the reader to rewrite all results in terms of G_0, G_1 into the experimentally relevant variables Q_0, Q_1.

Optimal geometry design

We show a cross section with all length scales of the optimal geometry in Fig. 2. We have a total width of $W = 2$mm, inner radius $R_2 = R_{\rm fiber} = 250\mu$m, and $W_{\rm inlet} = 0.2$mm. We chose for a distance between the inlet and the top and side of the flow channel of, respectively, $L_1 = 0.2$mm and $L_2 = 0.15$mm. The distance between the needle that supports the fiber and the inlets is $\Delta_{\rm needle,inlets} = 0.15$mm. This gives $R_3 = R_{\rm needle} + \Delta_{\rm needle,inlets} = 0.55{\rm mm} + 0.15{\rm mm} = 0.7$mm. Furthermore, we have a distance of $2 \cdot L_3$ between the two inlets, with $L_3 = L_2/2$. The area was minimized for this particular inlet structure, which gave for the height of the geometry $H = 0.218$mm. Finally, we have an outer radius of $R_1 = R_3 + W_{\rm inlet} + L_1 = 1.1$mm.

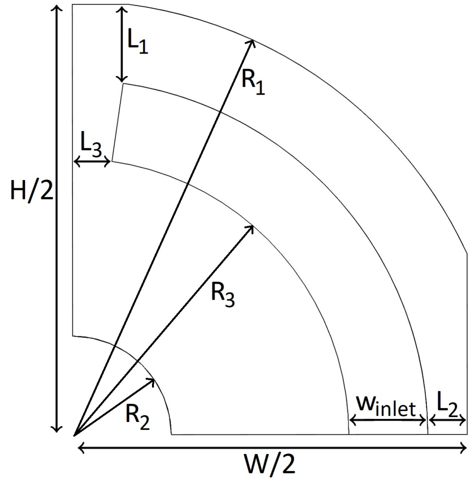

Figure 2: Cross section of the optimal geometry, showing all dimensions of the system.

Notes and references

[1] Nicky Langerak. Optimization of fluid flow in a novel organ chip. Master's thesis, Utrecht University, the Netherlands, 2019.

[2] L Gary Leal. *Advanced transport phenomena: fluid mechanics and convective transport processes*, volume 7. Cambridge University Press, 2007.
